# Effect of Previous-Winter Mortality on the Association between Summer Temperature and Mortality in South Korea

**DOI:** 10.1289/ehp.1002080

**Published:** 2011-01-13

**Authors:** Jongsik Ha, Ho Kim, Shakoor Hajat

**Affiliations:** 1 Korea Environment Institute, Seoul, South Korea; 2 Department of Biostatistics and Epidemiology, School of Public Health and the Institute of Health and Environment, Seoul National University, Seoul, South Korea; 3 Public and Environmental Health Research Unit, London School of Hygiene and Tropical Medicine, London, United Kingdom

**Keywords:** high temperature, mortality, preventive heath services, South Korea, weather

## Abstract

**Background:**

It has recently been postulated that low mortality levels in the previous winter may increase the proportion of vulnerable individuals in the pool of people at risk of heat-related death during the summer months.

**Objectives:**

We explored the sensitivity of heat-related mortality in summer (June–August) to mortality in the previous winter (December–February) in Seoul, Daegu, and Incheon in South Korea, from 1992 through 2007, excluding the summer of 1994.

**Methods:**

Poisson regression models adapted for time-series data were used to estimate associations between a 1°C increase in average summer temperature (on the same day and the previous day) above thresholds specific for city, age, and cause of death, and daily mortality counts. Effects were estimated separately for summers preceded by winters with low and high mortality, with adjustment for secular trends.

**Results:**

Temperatures above city-specific thresholds were associated with increased mortality in all three cities. Associations were stronger in summers preceded by winters with low versus high mortality levels for all nonaccidental deaths and, to a lesser extent, among persons ≥ 65 years of age. Effect modification by previous-winter mortality was not evident when we restricted deaths to cardiovascular disease outcomes in Seoul.

**Conclusions:**

Our results suggest that low winter all-cause mortality leads to higher mortality during the next summer. Evidence of a relation between increased summer heat-related mortality and previous wintertime deaths has the potential to inform public health efforts to mitigate effects of hot weather.

The association between elevated temperatures and mortality has been reported since the early 20th century. For example, Gover (1938) reported excess deaths associated with elevated ambient temperature exposure in 86 U.S. cities from 1925 to 1937. Studies of army recruits published in the 1940s (Schickele 1947) and 1950s (Stallones et al. 1957) also described an association between ambient heat exposure and mortality. Heat is undeniably a natural hazard and can have a pronounced effect on human health.

Concerns about the public health threat of elevated temperatures have increased in recent years, especially considering the potential impacts of climate change and the increased heat island effects in urban settings. The Intergovernmental Panel on Climate Change (2007) reported that climate change is likely to lead to more intense and frequent extreme weather events and that human exposure to such changing weather patterns could result in increased deaths, diseases, and suffering.

To understand the public health implications of heat effects, it is important to estimate the extent to which heat-related deaths in already frail individuals are simply hastened by heat exposure, a phenomenon referred to as mortality displacement (or “harvesting”) (Kovats and Hajat 2008). This process has been shown to occur on a short-term basis (within days or weeks of heat exposure) (Braga et al. 2001; Hajat et al. 2005; Kalkstein and Greene 1997; Kyselý and Kríz 2008), but evidence that summertime deaths after the Paris heat wave in 2003 may have influenced mortality the next winter suggest longer term effects as well (Valleron and Boumendil 2004). Only two previous studies have analytically assessed the association between mortality during winter and mortality during the next summer (Rocklöv et al. 2008; Stafoggia et al. 2009), and both reported that low levels of winter mortality were associated with higher estimated temperature effects the next summer.

We previously reported that mortality increases with temperatures above city-specific thresholds during the hot season in six major South Korean cities (Kim et al. 2006). The aim of this study was to assess the extent to which the association between summer (June–August) temperatures and mortality is modified by the mortality level of the previous winter (December–February).

## Materials and Methods

### Scope of the study

The cities selected for this study were Seoul, Daegu, and Incheon, South Korea. The study period ranged from 1992 through 2007, with the exception of the summer of 1994, which was excluded because of unusually hot weather. Summer and winter were defined as June–August and December–February, respectively. We examined deaths that occurred in all ages and among persons ≥ 65 years of age.

### Weather and mortality data

Measurements of relative humidity and ambient temperature taken every 3 hr were obtained from the Korea Meteorological Administration for the period 1992–2007. Daily mean temperature and humidity were calculated as the average of the 3-hr measurements from one representative meteorological station in each city. No important changes in station locations occurred during the study period, and each station recorded complete data series for the period 1992–2007. Based on previous work (Kim et al. 2006), we estimated associations between daily mortality counts and the average daily mean temperature during the same day and the previous day (referred to hereafter as a 0- to 1-day lag).

The Korea National Statistical Office provided daily mortality counts. Deaths of individuals who were not residents of the study area and accidental deaths [*International Classification of Diseases, 10th Revision* (ICD-10), codes V00–Y99] (World Health Organization 1993) were excluded. We estimated associations with all other causes of mortality (ICD-10, codes A00–U99) in all three study areas and estimated associations with cardiovascular disease (CVD)-related mortality (ICD-10, codes I00–I99) in Seoul only (because of limited power due to relatively small numbers of CVD-related deaths per day in Daegu and Incheon).

### Modeling approach

Poisson regression models adapted for time-series data were used to estimate short-term temperature effects on mortality levels. To control for intrasummer seasonal patterns, we included in the all models natural cubic spline (NCS) functions with 3 degrees of freedom (df) for summer date (i.e., from day 1 to day 91 of the summer season). Long-term temporal trends were accounted for by modeling indicator terms for each year (14 terms for 15 years). Indicator terms were also used to control for day of week and holiday effects—one term for the reference and three terms for the four categories of day of week and for holidays, including Sunday, the day after a holiday or holidays, Saturday, and other days. Average daily humidity on the current and previous day (0- to 1-day lag) was modeled using NCS (with 4 df).

We used three different models to estimate temperature effects. The first modeled mean temperature (0- to 1-day lag) used NCS (with 4 df) with adjustment for day of week and holiday, calendar year, summer date, and humidity (as described above) to assess the functional form of the temperature–mortality relationship:


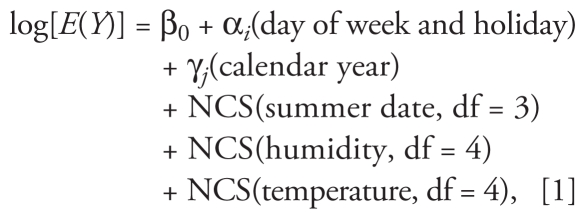


where *E*(*Y*) denotes expected daily death counts, and the subscript *i* refers to 1, 2, 3 for day of week and holiday; the subscript *j* refers to 1, 2, . . ., 14 for calendar year.

To quantify the temperature effect, temperature was modeled as a log-linear term that assumed no association below city-, age-, and cause-specific threshold temperature values and a linear increase in mortality above the threshold:


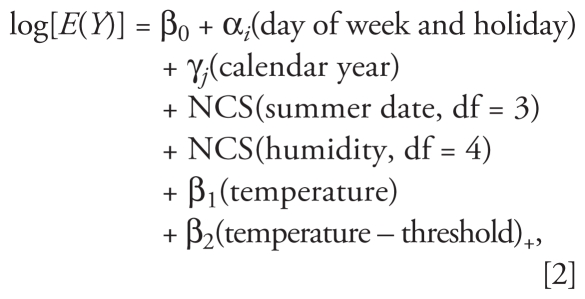


where (temperature – threshold)_+_ refers to max{temperature – threshold, 0} [i.e., 0 if the average temperature (0- to 1-day lag) was less than the city-, age-, and cause-specific threshold value].

Temperature threshold values used in this model were determined based on the best fitting model [as determined by Akaike’s information criterion (Akaike 1973)] among models that used different threshold values (in 0.1°C increments of potential threshold values based on graphical inspection). The precision of the threshold estimate for each series was determined using a resampling technique in which the models used to identify the optimum threshold were repeated 1,000 times with 10% of the subjects omitted at random from each iteration. This procedure for temperature threshold values was applied to each city, age, and cause of death stratum.

A third model was used to estimate the effect of previous-winter mortality on the association between temperature and mortality in the summer months:


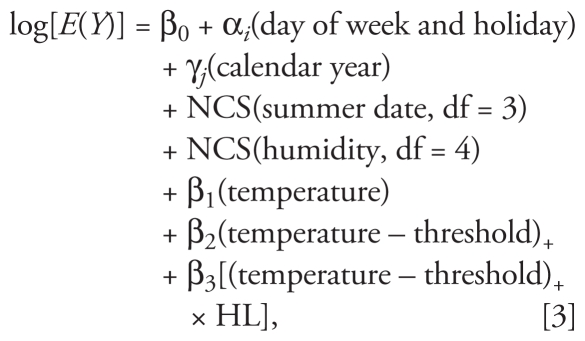


where HL refers to high–low mortality. To classify winters as having high or low mortality, we regressed yearly average winter mortality against time (i.e., calendar year) to determine predicted mortality after accounting for secular trends according to city and age (all or ≥ 65) for all nonaccidental deaths. Winters with observed mortality counts above the model prediction were classified as high-mortality winters (HL = 0), and those with observed mortality counts below the model predicted value were classified as low-mortality winters (HL = 1; see Table 1). Summer temperature (0- to 1-day lag) was modeled as a log-linear term assuming as common threshold temperature value for each city across all years combined, because summer temperature thresholds for years with high winter mortality (27.9°C, 28.8°C, and 26.8°C for Seoul, Daegu, and Incheon, respectively) fell within the 95% confidence intervals (CIs) of thresholds estimated for years with low winter mortality (27.3°C, 27.6°C, and 25.8°C for Seoul, Daegu, and Incheon, respectively) and vice versa. An interaction term for winter mortality (HL = 0, 1) and summer temperature (0- to 1-day lag) above the city-, age-, and cause-specific threshold value was used to determine whether mortality during the previous winter modified the association between mortality and summer temperature.

**Figure 1:**
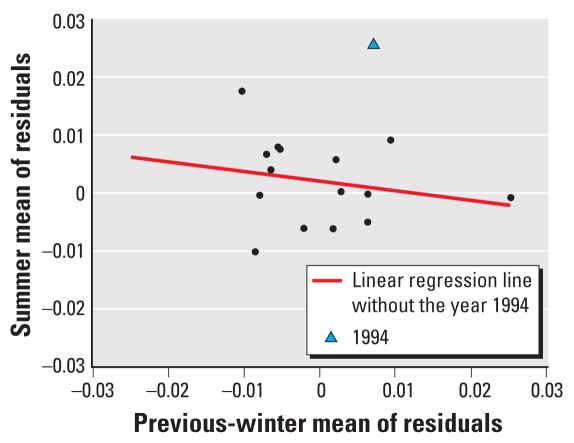
Summer (June–August) mean of residuals versus previous-winter (December–February) mean of residuals for all nonaccidental deaths in Seoul, from 1992 to 2007, by year, estimated using a Poisson regression model adjusted for date using NCS (with 64 df) and for calendar year, season (one term for the reference and three terms for four seasons: March–May, June–August, September–November, and December–February), and day of week and holiday using indicator variables. Correlation coefficient for the 15 year-specific estimates (excluding 1994) is −0.2096 (*p* = 0.453).

We conducted several sensitivity analyses to check the robustness of our findings. First, we evaluated effect modification by four versus two categories of winter mortality. Second, we evaluated the effect of previous-spring mortality burden on the association between summer temperature and mortality for all-ages deaths from natural causes. Third, we evaluated the effect of winter mortality burden in the winter before the most recent winter (e.g., modification of heat-related mortality in the summer of 2001 by winter mortality in the winter of 1999–2000). Finally, heat waves in most summers have been positively associated with mortality, but the heat wave from July to August 1994 (Figure 1) had a particularly high impact on mortality (Choi et al. 2005; Kyselý and Kim 2009). Because the 1994 heat wave might have been an extremely rare event, we excluded 1994 data from the analysis when evaluating modification of the summertime temperature–mortality relationship by previous-winter mortality levels. However, we also conducted analyses with 1994 data included to assess the potential impact of this event.

All analyses were performed using S-Plus 2000 (Mathsoft Inc., Cambridge, MA, USA). The convergence tolerances of the regression models were set to 10^−9^, with a limit of 1,000 iterations to avoid biased estimates of regression coefficients and standard errors (Dominici et al. 2002; Pattenden et al. 2003).

## Results

### Description

**Figure 2:**
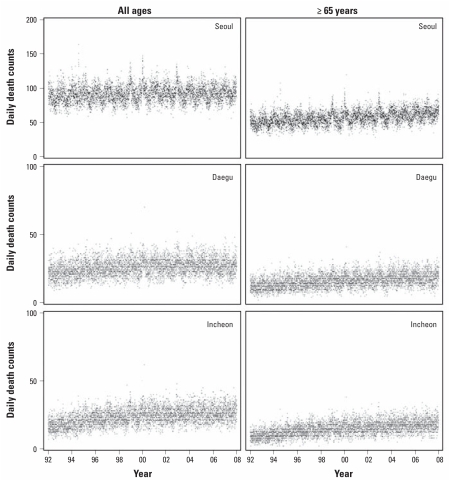
Daily death counts for all ages and the ≥ 65 year age group in Seoul, Daegu, and Incheon, 1992–2007.

The time series of daily death counts from 1992 through 2007 show a clear annual pattern in daily death counts, with peaks in winter and dips in summer, and an increasing trend in numbers of deaths during the study period (Figure 2). Average summer temperatures over the entire study period were 24.21°C, 25.11°C, and 23.54°C in Seoul, Daegu, and Incheon, respectively. However, average temperatures in summer 1994 were exceptionally high in all three cities (26.3°C, 27.5°C, and 25.2°C, respectively; Table 1).

Figure 1 shows the correlation between annual summer mortality and previous wintertime mortality from all nonaccidental causes of death (Seoul, all ages). Although not statistically significant, summer mortality was inversely associated with mortality in the previous winter.

**Figure 3:**
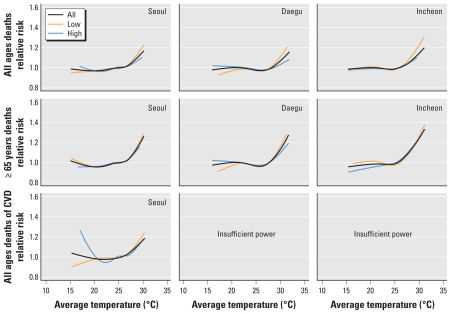
Combined (all cities studied) exposure–response curve for mean daily temperature (0- to 1-day lag) and daily death counts (all nonaccidental deaths and CVD deaths) for all ages and among those ≥ 65 years of age in summer (June–August), 1992–2007 (excluding 1994), with and without stratification by the previous-winter mortality level (high or low).

Figure 3 displays the exposure–response relationship between the moving average (lag 0–1) of daily mean summer temperature and daily death count for all summers combined and for summers stratified by low or high previous-winter mortality. All plots in the graphical analysis exhibited a rapidly increasing pattern of the risk of mortality as temperature increases above the threshold. For all summers combined, associations with higher temperatures appeared stronger *a*) for the ≥ 65 year age group than for all ages combined, *b*) for CVD-related mortality than for all-cause–related mortality (all ages combined) in Seoul, and *c*) for Seoul than for the other cities. Results for Seoul also suggest an effect of relatively low summer temperatures on CVD risk. Associations with higher summer temperatures were stronger after low-mortality winters than after high-mortality winters for all ages combined and, to a lesser extent, for deaths among persons ≥ 65 years of age.

### Quantification of effects

From model 2 (see Equation 2) for all summer data, the summer temperature thresholds (0- to 1-day lag) for all nonaccidental causes of death were 27.9°C, 28.1°C, and 25.5°C for all ages combined and 27.4°C, 27.3°C, and 24.1°C for those ≥ 65 years of age in Seoul, Daegu, and Incheon, respectively (Table 2). According to model 2 estimates, 1°C increases in summer temperatures (0- to 1-day lag) above the thresholds were associated with increases in mortality of 7.97% (95% CI, 5.50–10.49%), 6.12% (3.74–8.55%), and 3.85% (1.80–5.94%) for all ages combined, and 8.51% (6.26–10.81%), 6.82% (4.56–9.13%), and 3.89% (2.12–5.69%) among those ≥ 65 years of age in Seoul, Daegu, and Incheon, respectively (Table 2). A 1°C increase in summer temperatures above a threshold of 27.9°C was associated with a 10.16% (5.36–15.18%) increase in CVD-related mortality in Seoul (all ages combined). All effect estimates were statistically significant at *p* < 0.05.

Based on model 3 estimates (see Equation 3) for all nonaccidental deaths in all age groups, a 1°C increment in summer temperature above the thresholds was associated with increases of 10.57%, 8.55%, and 6.04% in summer mortality after a low-mortality winter and 4.85%, 4.00%, and 2.63% increases in summer mortality after a high-mortality winter in Seoul, Daegu, and Incheon, respectively (*p*-values for effect modification by a previous high- vs. low-mortality winter of 0.008, 0.014, and 0.038, respectively; Table 2). In the ≥ 65 years age group, summer mortality increased by 8.85%, 8.36%, and 4.78% in association with a 1°C increase in summer temperature above threshold values after a low-mortality winter and by 7.75%, 5.14%, and 3.32% after a high-mortality winter in Seoul, Daegu, and Incheon, respectively (*p*-values for effect modification of 0.585, 0.050, and 0.226, respectively). CVD-related mortality (all ages) in Seoul was comparable after low- and high-mortality winters (Table 2).

Separating winters into four groups based on mortality levels limited our power to estimate effects, but results suggested that the broad pattern of higher heat risk after low-mortality winters remained (data not shown). We did not find evidence that mortality in the previous spring modified the association between temperature and mortality in the next summer (*p*-values for effect modification in Seoul, Daegu, and Incheon of 0.946, 0.578, and 0.338, respectively) or that mortality two winters prior modified the association (*p*-values for interaction terms in Seoul and Daegu of 0.459 and 0.804, respectively). Finally, estimated effects for summer heat-related mortality after a low-mortality winter were still larger after a high-mortality winter when we included data from 1994 in analyses (estimated increases in all nonaccidental deaths among all ages of 11.03%, 7.91%, and 7.14% for low previous-winter mortality and 10.42%, 4.19%, and 2.79% for high previous-winter mortality in Seoul, Daegu, and Incheon, respectively).

## Discussion

The results of this study confirm previous findings that summer heat exposure is an important predictor of death in South Korea, particularly in the capital city of Seoul (Kim et al. 2006). The more novel finding of this study is that the risk of heat-related death in the summer was higher when we classified the preceding wintertime mortality burden as low versus high. This observation is consistent with the hypothesis that a winter with relatively low mortality level leaves a larger pool of people susceptible to heat-related mortality in the next summer. Conversely, a high winter mortality burden may reduce the number of people at risk of heat-related mortality in subsequent months. Our findings are consistent with a process of forward mortality displacement (or “harvesting”), with initial increases in mortality (mostly in already frail individuals) that are followed by deficits in expected number of deaths sometime after the initial excess. Time-series regression studies have shown that, on a short-term basis (i.e., over a few days or weeks), some mortality associated with heat exposure can be attributed to this displacement process, but there appears to be less of an effect on cold-related mortality (Braga et al. 2001). Displacement occurring over a longer time frame (i.e., months) is more difficult to assess because of the need to control for seasonal patterns.

Our study, initially looking at year-to-year correlations, indicates that some wintertime deaths may indeed be displaced over a longer term, to the extent that mortality in the next summer may be less than expected. Although such a possibility has been postulated before (Fouillet et al. 2008), only two previous studies have demonstrated this analytically (Rocklöv et al. 2008; Stafoggia et al. 2009). As with our work, both of these studies were conducted in high-income settings (i.e., where temperature-related deaths were dominated by chronic diseases in the elderly), with both indicating a greater heat risk in summers after a low wintertime burden. We observed this pattern occurring with all-cause mortality for all ages and, to a lesser extent, among the elderly (age ≥ 65 years). The fact that the difference in heat risk between high and low winter mortality years was larger in the all-ages group than in the elderly is counterintuitive if the process is truly reflecting long-term mortality displacement of the most frail individuals. Future studies stratified by both age- and cause-specific death groups in larger samples, and possibly studies that also account for specific heat-wave periods and their duration, may clarify whether our findings are the result of such harvesting processes. In contrast to the two previous studies (Rocklöv et al. 2008; Stafoggia et al. 2009), we did not observe similar patterns for all-cause and CVD deaths, but we were able to assess CVD-related mortality in one city where power was sufficient to estimate associations with daily death counts. Previous studies have shown that, on a short-term basis, displacement is more strongly associated with heat-related CVD deaths than with deaths from other causes, including respiratory diseases (Goodman et al. 2004; Hajat et al. 2005). Our findings suggest that a displacement effect for non-CVD deaths does indeed occur but may become apparent only over a longer time scale.

Rocklöv et al. (2008) observed that influenza epidemics may contribute to a low subsequent summertime mortality, but we were unable to evaluate the effect of the influenza epidemic on the association between summer temperature and mortality because influenza data are available only at the national (vs. city) level.

Another potential limitation of our analysis is that we were unable to control for air pollution for the full period analyzed, although controlling for ozone did not affect the modification by mortality in the previous winter in the study conducted in Rome (Stafoggia et al. 2009).

The existence of threshold effects means that the risk estimates based on an assumption of linearity may underestimate mortality risks due to elevated temperatures (Kim et al. 2004). We used common temperature thresholds across all years to facilitate estimation of the main parameter of interest: the heat slope. However, it is possible that high winter mortality may reduce the temperature threshold for summer heat-related mortality by weakening individuals and therefore heightening their subsequent heat risk, or that high winter mortality may raise the temperature threshold by increasing the proportion of healthy subjects that are less susceptible to heat, thereby increasing the threshold temperature required for a heat effect to become apparent.

Sensitivity analyses suggested that effect modification by previous-winter mortality persisted when we classified winter mortality into four (vs. two) groups, although power was limited to estimate stratum-specific effects. In addition, modification was evident when we included data from 1994 (including deaths during the heat wave from July to August of that year) in analyses. However, we did not find evidence that mortality in the previous spring or in the winter 2 years prior modified heat-related mortality in the summer.

Mirroring many countries globally, South Korea has had a heat/health watch warning system in operation since 2007 to protect human health from the dangers of hot weather. Because monthly mortality data in South Korea are available only after a 3-month delay, information on the number of wintertime deaths could, in an operational sense, be used to estimate early information on expected summertime burdens. Although long-term weather forecasts are often used by some countries as part of their heat plans to prepare for expected summertime activity levels, useful information may also be provided by information on wintertime mortality levels in settings where our findings are replicated.

## Conclusions

The results of our study indicate that increased summer heat-related mortality is associated with previous wintertime deaths. We recommend that public health strategies to minimize adverse health impacts of heat waves, including the South Korean heat/health watch warning system, should account for potential effects of mortality in the previous winter.

## Figures and Tables

**Table 1:** Average daily number of deaths and temperature in the winter and summer seasons in Seoul, Daegu, and Incheon, 1992–2007.

City	Winter (December–February)			Summer (June–August)				Temperature (°C)^b^
	Year	Deaths^a^		Year	Deaths			
		All	≥ 65 years		All^a^	CVD related	≥ 65 years^a^	
Seoul	1992	92.7 (L)	54.3 (H)	1992	80.8	26.5	45.4	23.5
	1992/1993	91.5 (L)	53.4 (L)	1993	81.8	25.3	46.2	23.5
	1993/1994	96.9 (H)	57.4 (H)	1994^c^	91.9	28.4	54.3	26.3
	1994/1995	96.8 (H)	57.3 (H)	1995	82.8	22.8	47.9	24.1
	1995/1996	93.0 (L)	55.2 (L)	1996	84.3	23.8	50.0	24.2
	1996/1997	95.4 (H)	57.0 (L)	1997	85.3	22.1	51.7	25.5
	1997/1998	93.3 (L)	56.1 (L)	1998	85.3	22.1	51.6	24.0
	1998/1999	104.7 (H)	65.2 (H)	1999	84.8	20.7	50.3	25.0
	1999/2000	108.8 (H)	68.1 (H)	2000	87.6	21.5	52.7	25.6
	2000/2001	97.2 (H)	60.2 (L)	2001	88.5	22.9	54.9	25.0
	2001/2002	95.3 (L)	59.2 (L)	2002	87.4	24.0	54.0	23.9
	2002/2003	102.1 (H)	66.8 (H)	2003	84.3	23.0	52.8	23.2
	2003/2004	96.1 (L)	63.4 (L)	2004	86.8	24.4	57.4	24.7
	2004/2005	95.9 (L)	63.4 (L)	2005	85.6	24.0	55.8	24.4
	2005/2006	96.8 (L)	66.4 (H)	2006	88.8	24.9	60.1	24.0
	2006/2007	99.2 (H)	68.5 (H)	2007	87.1	23.3	59.8	24.6
Daegu	1992	22.5 (L)	12.8 (L)	1992	20.4	6.2	11.2	25.0
	1992/1993	23.8 (L)	13.4 (L)	1993	21.2	5.9	12.1	22.9
	1993/1994	24.1 (L)	13.5 (L)	1994^c^	23.5	7.4	13.6	27.5
	1994/1995	25.6 (H)	15.2 (H)	1995	24.1	6.7	13.8	25.5
	1995/1996	26.2 (H)	15.6 (H)	1996	24.3	6.3	14.0	24.9
	1996/1997	27.3 (H)	16.5 (H)	1997	25.0	5.7	14.4	25.5
	1997/1998	26.7 (H)	16.6 (H)	1998	25.0	6.1	14.9	24.1
	1998/1999	28.7 (H)	18.8 (H)	1999	25.5	6.3	15.2	24.5
	1999/2000	31.1 (H)	20.0 (H)	2000	24.5	5.9	14.5	25.6
	2000/2001	29.1 (H)	18.6 (H)	2001	25.5	6.5	15.8	26.3
	2001/2002	28.2 (L)	18.2 (L)	2002	25.4	7.1	16.0	24.8
	2002/2003	31.5 (H)	20.8 (H)	2003	24.6	6.6	15.8	23.3
	2003/2004	29.0 (L)	19.5 (L)	2004	26.9	6.8	17.8	25.4
	2004/2005	29.6 (L)	20.3 (L)	2005	25.3	6.5	17.2	25.9
	2005/2006	29.3 (L)	20.4 (L)	2006	25.5	7.1	17.3	25.3
	2006/2007	29.4 (L)	20.7 (L)	2007	24.4	6.8	16.8	25.2
Incheon	1992	17.1 (L)	9.1 (L)	1992	16.0	5.6	9.0	22.6
	1992/1993	19.1 (L)	10.5 (L)	1993	16.4	5.4	8.9	21.8
	1993/1994	19.9 (L)	11.5 (L)	1994^c^	19.9	6.5	11.3	25.2
	1994/1995	22.3 (H)	12.7 (H)	1995	20.1	6.0	11.8	23.2
	1995/1996	23.5 (H)	14.1 (H)	1996	21.2	5.9	12.4	23.2
	1996/1997	24.9 (H)	15.2 (H)	1997	22.1	5.7	13.2	24.3
	1997/1998	24.8 (H)	15.1 (H)	1998	22.3	6.6	13.7	23.2
	1998/1999	27.3 (H)	17.0 (H)	1999	22.6	6.0	14.0	23.9
	1999/2000	27.6 (H)	17.7 (H)	2000	24.4	6.5	15.0	25.0
	2000/2001	26.8 (H)	16.7 (H)	2001	23.5	7.1	14.4	24.7
	2001/2002	25.2 (L)	16.4 (L)	2002	23.5	7.1	14.3	23.9
	2002/2003	27.3 (H)	18.2 (H)	2003	25.4	7.4	16.5	22.3
	2003/2004	27.5 (L)	17.8 (L)	2004	24.6	6.9	16.4	23.7
	2004/2005	28.0 (L)	18.5 (L)	2005	24.5	6.6	16.3	23.4
	2005/2006	27.4 (L)	18.9 (L)	2006	25.7	7.6	16.9	23.0
	2006/2007	28.2 (L)	19.3 (L)	2007	26.2	7.4	17.6	23.4

Abbreviations: H, high previous-winter mortality level; L, low previous-winter mortality level.

a All nonaccidental deaths.

b Mean daily temperature (0-lag day).

c The 1994 data were not used in any other analyses.

**Table 2:** Summer temperature thresholds and estimated increase in mortality (95% CI) associated with a 1°C increase in temperature above the thresholds in Seoul, Daegu, and Incheon, South Korea.

City and age group	Observed threshold^b^ (°C)	Percentage increase above the threshold^a^			*p*-Value^c^
		All data	Winter mortality		
			High	Low	
Deaths from natural causes					

Seoul					
All ages	27.9 (27.02–28.28)	7.97 (5.50–10.49)	4.85 (1.54–8.26)	10.57 (7.30–13.93)	0.008
≥ 65 years	27.4 (27.07–28.37)	8.51 (6.26–10.81)	7.75 (4.12–11.41)	8.85 (6.22–11.53)	0.585
Daegu					
All ages	28.1 (27.05–29.01)	6.12 (3.74–8.55)	4.00 (1.00–7.08)	8.55 (5.47–11.73)	0.014
≥ 65 years	27.3 (26.61–28.42)	6.82 (4.56–9.13)	5.14 (2.28–8.08)	8.36 (5.59–11.21)	0.050
Incheon					
All ages	25.5 (25.28–28.47)	3.85 (1.80–5.94)	2.63 (0.26–5.05)	6.04 (3.00–9.17)	0.038
≥ 65 years	24.1 (22.84–27.91)	3.89 (2.12–5.69)	3.32 (1.29–5.39)	4.78 (2.42–7.19)	0.226

Deaths from CVD^d^					

All ages	27.9 (26.69–28.47)	10.16 (5.36–15.18)	10.07 (3.35–17.22)	10.23 (4.05–16.77)	0.968

a Percentage increase in daily mortality with a 1°C temperature increase above the threshold.

b The temperature at which the risk of mortality begins to increase with increasing temperature.

c *p*-Value for interaction term between temperature above the threshold value and previous-winter mortality (high or low).

d Estimated associations with CVD-related mortality for Seoul, South Korea, only.
